# Assessment of cognitive functioning using the Mini Mental State Examination in men with alcohol and crack cocaine use disorder

**DOI:** 10.47626/2237-6089-2022-0567

**Published:** 2023-08-04

**Authors:** Jaqueline B. Schuch, Natália Becker, Francisco Diego Rabelo-da-Ponte, Felipe Ornell, Hellen J. M. Freitas, Fernando P. Rebelatto, Anne O. Sordi, Flavio Pechansky, Lisia von Diemen, Felix H. P. Kessler

**Affiliations:** 1 Centro Colaborador em Álcool e Drogas Hospital de Clínicas de Porto Alegre Universidade Federal do Rio Grande do Sul Porto Alegre RS Brazil Centro de Pesquisa em Álcool e Drogas, Centro Colaborador em Álcool e Drogas, Hospital de Clínicas de Porto Alegre, Universidade Federal do Rio Grande do Sul (UFRGS), Porto Alegre, RS, Brazil.; 2 UFRGS Porto Alegre RS Brazil Programa de Pós-Graduação em Psiquiatria e Ciências do Comportamento, UFRGS, Porto Alegre, RS, Brazil.; 3 Universidade Presbiteriana Mackenzie São Paulo SP Brazil Programa de Pós-Graduação em Distúrbios do Desenvolvimento, Universidade Presbiteriana Mackenzie, São Paulo, SP, Brazil.; 4 Social, Genetic and Developmental Psychiatry Centre Institute of Psychiatry, Psychology & Neuroscience King’s College London London UK Social, Genetic and Developmental Psychiatry Centre, Institute of Psychiatry, Psychology & Neuroscience, King’s College London, London, UK.

**Keywords:** Substance-related disorders, language comprehension, alcohol-related disorders, cocaine, cocaine smoking

## Abstract

**Introduction:**

Consumption of substances has been associated with cognitive impairment. The Mini Mental State Examination (MMSE) is an easy-to-apply screening tool used to assess cognitive functions.

**Objectives:**

To evaluate the cognitive performance of individuals with alcohol (AUD) and/or crack cocaine use disorder (CUD) and polysubstance use using the MMSE and to investigate the impact of substance use profile and the moderation effect of educational level on MMSE performance.

**Methods:**

Cross-sectional study with 508 adult male inpatients diagnosed with substance use disorders (245 with AUD, 85 with CUD, and 178 with polysubstance use). Cognitive performance was assessed using the MMSE scale (total and composite scores).

**Results:**

Individuals with AUD had worse total MMSE scores and scored worse for all three MMSE components compared to individuals with polysubstance use (p < 0.001, oral/written language comprehension, p < 0.001, attention/memory, and p = 0.007, motor functions). MMSE scores were positively correlated with educational level (p < 0.017), but were not associated with age, recent drug use, or years of drug use. Educational level moderated the impact of substance use on MMSE performance, especially total score and composite language comprehension score. Individuals with a low educational level (≤ 8 years) had worse performance than those with a high educational level (≥ 9 years), mainly in individuals with AUD (p < 0.001).

**Discussion:**

Individuals with a low educational level and alcohol use are more prone to present cognitive impairment than crack cocaine users, especially involving language aspects. Better-preserved cognitive function could impact treatment adherence and might guide the decision of therapeutic strategies.

## Introduction

Substance use disorders (SUD) involve the compulsive use of substances, and may lead to physical harm and problems in a wide array of physiological and cognitive domains.^1^ Alcohol is the most prevalent substance of abuse, and approximately 43% of the world population are current drinkers.^2^ In the Americas region, the prevalence of alcohol use disorder (AUD) ranges from 5.1 to 11.5%.^2^ The overall prevalence of use of other substances is 5.4%, including cannabis (4%), opioids (1.2%), and cocaine (0.4%).^3^ Approximately 36.3 million people suffer from SUD (not including AUD), which corresponds to 0.7% of the worldwide population.^3^

Among individuals with SUD, the prevalence of cognitive impairments is heterogeneous – ranging from 30 to 80%, depending on aspects such as the substance used, period and pattern of consumption, and sociodemographic variables.^4 , 5^ Chronic alcohol use has been related to impairments in attention, language, fluency, processing speed, memory, working memory, and decision-making.^4 , 6 - 9^ Moreover, deficits in verbal learning, visual and verbal memory, and processing speed might persist even after abstinence for 1 year or more,^7^ suggesting a sustained and deleterious effect of alcohol use on cognitive performance. Cocaine users also present deficits in inhibition, mental flexibility, attention, learning, verbal working memory, and decision-making processes.^6^ However, the long-term effects of cocaine use on cognition are not as fully elucidated as those caused by alcohol.^10^ The use of multiple substances, including the simultaneous use of alcohol and cocaine, has been associated with deficits in working memory, cognitive flexibility, and inhibitory control.^11^ Nevertheless, the effect and the contribution of specific substances are difficult to disentangle among subjects with polysubstance use. Overall, individuals with SUD are more prone to deficits in global cognitive ability – particularly in attention, memory, verbal and visuospatial abilities, inhibition, working memory, and decision-making, and also in psychomotor abilities.^12 - 14^

Comprehensive neuropsychological assessment of individuals with SUD during treatment is a complex task, requiring multiple interviews and evaluation sessions.^12^ However, implementation of brief cognitive screenings capable of identifying potential cognitive impairments is crucial. The Mini Mental State Examination (MMSE) is widely used as a screening tool and assesses different cognitive components, such as orientation, memory, attention, calculation, and language abilities.^15 , 16^ This test may indicate the severity, or the deleterious effect on cognition of a given substance, allowing identification of the most affected cognitive function in each case. Lower MMSE scores compared to controls have been observed among alcohol, crack cocaine, opioid, and methamphetamine users.^17 - 19^ A previous study also demonstrated that MMSE could be used as a cognitive screening tool and as a criterion for recruiting individuals with SUD who present greater adherence in clinical trials.^20^ In this sense, cognitive deficits may have a negative impact on several clinical outcomes, including treatment retention and adherence in individuals with alcohol addiction^21^ and cocaine and amphetamine dependence.^22^ More effective interventions such as pharmacotherapy and psychosocial interventions with contingency management,^23 , 24^ as well as the implementation of abstinence-sustaining strategies,^25^ require better preserved cognitive capacity for improved outcomes.^14 , 19^

Besides the addiction itself, educational level and age could also affect performance in cognitive assessments in different populations.^26 , 27^ Among individuals with SUD, evidence shows that education strongly contributes to cognitive performance, being correlated with verbal measures – such as vocabulary and abstraction.^13^

Considering that different substances of abuse might have effects on specific cognitive functions, our aim was to investigate the MMSE performance of individuals with SUD according to the substance used (alcohol, crack cocaine, or polysubstance use). Our hypothesis was that individuals with AUD (alcohol only and polysubstance use – in combination with other substances) would perform worse in the MMSE than individuals with crack cocaine use disorder. We also analyzed the possible moderating effect of educational level on MMSE performance. Educational level could moderate cognitive performance impairment triggered by substance use. Lastly, we aimed to evaluate the possible relationships between total MMSE score and characteristics related to substance use, including years of use, use in the last 30 days, and severity of addiction, days in hospital, and presence of psychiatric comorbidities.

## Methods

### Sample

This is a cross-sectional study using a secondary database comprising 508 male inpatients from a detoxification unit at a public hospital in Southern Brazil (from February 2017 to October 2019). All individuals were diagnosed with SUD according to Diagnostic and Statistical Manual of Mental Disorders, 5th edition (DSM-5) criteria.^28^ This convenience sample included 245 individuals with AUD, 85 individuals with cocaine use disorder (CUD) (including individuals with crack and/or cocaine use disorder), and 178 individuals with polysubstance use (individuals with alcohol and crack/cocaine use disorder). Inclusion criteria consisted of a diagnosis of SUD, male sex, and age of 18 years or more. Participants were excluded from the study if they had clinical, cognitive, or psychiatric conditions that could interfere with understanding of and, consequently, answers to the questionnaires, including presence of psychotic symptoms, other severe psychiatric symptoms, and severe medical conditions (i.e., Wernicke encephalopathy) or if they were disoriented inpatients.

This study was approved by the Ethics Committee at the Hospital de Clínicas de Porto Alegre (no. 2014-0249) and is in accordance with the Declaration of Helsinki guidelines. All individuals provided written informed consent.

### Research protocol

The research protocol was implemented during patients’ detoxification periods – between the 2nd and 6th day of hospitalization. It is important to mention that patients are admitted to the hospital having already spent 2 or 3 days in detoxification during the period they stay in the city’s psychiatric emergency room or at a psychosocial care center (Centro de Atenção Psicossocial [CAPS]). Interviews were therefore conducted after at least 4 or 5 days of detoxification, after stabilization of withdrawal symptoms. Individuals were interviewed by trained psychology undergraduate students under the supervision of a senior psychologist. All data were collected using the following instruments: a sociodemographic and clinical data questionnaire, the Addiction Severity Index 6th version (ASI-6), and the MMSE. Briefly, the ASI-6 is a semi-structured interview, validated for the Brazilian culture,^29^ that assesses different dimensions related to the severity of addiction: medical, employment status, legal aspects, family/social, psychiatric, use of alcohol, and use of other drugs. The MMSE is a cognitive screening tool that evaluates multiple cognitive aspects, including temporal and spatial orientation, episodic memory, auditory attention and arithmetic, oral and written language, and constructive praxis.^16 , 30^ It is considered a simple and standardized cognitive screening method, which is quick and easy to administer. The maximum MMSE score is 30 points and cut-off points vary depending on the sample being assessed.^15^ In our study, individuals with no formal education were included in the study if they could write their own name and understand the MMSE questions.

### Statistical analyses

The normality of continuous data distributions was tested using histograms and the Shapiro-Wilk test. Sociodemographic data, treatment retention, and drug use profile were compared between the three groups (AUD, CUD, and polysubstance use) using chi-square and Kruskal-Wallis tests. For categorical data, standardized adjusted residues were used to detect categories with higher than expected frequencies. The relationship between MMSE score and characteristics related to substance use (e.g., years of substance use, substance use in the last 30 days, substance use severity, and treatment retention [days in hospital]) were analyzed separately for each SUD group using Spearman correlations. We conducted a principal component analysis (PCA) with the purpose of grouping MMSE items into a series of components that would be able to tackle cognitive dimensions in inpatients with SUD. We used oblique rotation due to the high degree of correlation among items.

In order to verify our first hypothesis, we conducted Kruskal-Wallis tests to compare MMSE performance (total scores and components identified by PCA) according to substance use groups. For pairwise comparisons, Dunn-Bonferroni post-hoc tests were carried out and adjusted significance p-values were applied. A generalized linear model (GzLM) was also performed to analyze the possible moderating effect of educational level on MMSE performance (total scores and components produced by PCA). For this analysis, an interaction term (substance use versus educational level) was applied, and age was included as a covariate. Education status was divided into two groups: a) low educational level (LEL), consisting of individuals who had 0 to 8 formal years of education (n = 306); and b) high educational level (HEL), consisting of individuals who had 9 or more years of formal education (n = 202). The Bonferroni post-hoc test was used to account for multiple comparisons. For all analyses, a significance level of 5% and two-tailed tests were adopted.

## Results

Sociodemographic, clinical, and substance use data are shown in Table 1 . Individuals with AUD were older, with lower educational level, and stayed longer in hospital for detox. Individuals with CUD were younger, had shorter substance use time (in years), and lower severity scores compared to other groups of users. Use of other substances, such as cannabis, hallucinogens, and inhalants, was more prevalent among polysubstance users (p ≤ 0.001). Correlation analyses showed a medium to large positive correlation between MMSE score and years of formal education among individuals with AUD (p < 0.001), and polysubstance use (p < 0.001) ( Table 2 ). There was also a small significant positive correlation between total MMSE score and years of formal education (p = 0.017) ( Table 2 ) in individuals with CUD. Among individuals with low MMSE scores (< 18, n = 40), most were alcohol users (n = 26 [65%], p = 0.040) and had an LEL (n = 38 [95%], p < 0.001). Presence of major depressive disorder or generalized anxiety disorder did not impact total MMSE scores in the three groups of substances (p > 0.05).

**Table 1:** Sociodemographic, treatment retention, and substance use data

	AUD (n = 245)	CUD (n = 85)	Polysubstance use (n = 178)	p-value
Skin color (white)	145 (59.2)	50 (58.8)	95 (53.4)	0.461
Marital status (single)	169 (69.3)	65 (76.5)	125 (70.2)	0.443
Occupational status				< 0.001
Employed	74 (30.7)^-2.6^	33 (39.8)	75 (42.6)^2.1^	
Unemployed	85 (35.3)^-2.3^	44 (53.0)^2.5^	74 (42.0)	
Retired	82 (34.0)^5.7^	6 (7.2)^-3.7^	27 (15.3)^-3.0^	
Age (years)	51.54 (9.5)^a^	33.11 (9.6)^b^	41.28 (10.4)^c^	< 0.001
Educational level (years)	7.36 (3.6)^a^	8.02 (3.2)	8.64 (3.3)^b^	0.001
Days in hospital	30.45 (18.2)^a^	15.26 (15.9)^b^	20.43 (15.7)^c^	< 0.001
Years of substance use	26.13 (12.5)^a^	8.97 (6.3)^b^	20.68 (11.2)^c^	< 0.001
Use in the last 30 days	22.80 (10.0)^a^	13.18 (10.5)^b^	20.96 (10.8)^a^	< 0.001
Severity of drug use*	65.23 (7.7)^a^	54.34 (5.9)^b^	61.75 (8.5)^c^	< 0.001
Use of other drugs				
Cannabis	10 (4.1)^-8.5^	23 (27.1)	66 (37.1)^7.4^	< 0.001
Hallucinogens	0 (0.0)^-3.8^	4 (4.7)	11 (6.2)^3.2^	0.001
Inhalants	1 (0.4)^-4.2^	2 (2.4)	19 (10.7)^5.2^	< 0.001

Values are presented as mean (standard deviation) or n (%).

Kruskal-Wallis tests were performed to compare mean ranks between groups. Chi-square tests were performed to compare frequencies between groups.

Superscript letters show significant pairwise comparisons (p < 0.05).

Superscript numbers represent standardized adjusted residuals (> 1.96).

* Drug use severity in individuals with alcohol use disorder (AUD **)** was assessed using the Alcohol Addiction Severity Index (ASI) score; for cocaine/crack use disorder (CUD), these data were obtained from Drug ASI scores, and the higher value from Alcohol or Drug ASI score is presented for polysubstance use.

**Table 2:** Spearman correlations between Mini Mental State Examination (MMSE) score and age, educational level, treatment retention, and substance use data

	AUD	CUD	Polysubstance use
	
		MMSE score	
Age (years)	-0.042	-0.055	-0.063
Educational level (years)	0.534*	0.258^†^	0.460*
Days in hospital	0.007	0.149	0.046
Years of substance use	-0.093	-0.296	-0.089
Use in the last 30 days	-0.162	-0.307	-0.119
Severity of drug use^‡^	0.058	-0.292	-0.111

Values indicate Spearman coefficients.

* p < 0.001; ^†^ p < 0.017.

^‡^ Drug use severity in individuals with alcohol use disorder (AUD **)** was assessed using the Alcohol Addiction Severity Index (ASI) score; for cocaine/crack use disorder (CUD), these data were obtained from Drug ASI scores, and the higher value from Alcohol or Drug ASI score is presented for polysubstance use.

For the PCA, the Kaiser-Meyer-Olkin (KMO) value was 0.807 and Bartlett’s test of sphericity was statistically significant (χ^2^_[55]_ = 809.55, p < 0.001). It is recommended that KMO values should be higher than 0.6 and Bartlett’s test of sphericity should be significant. Since these conditions were met, we extracted three factors from the PCA analysis, which were defined as “oral/written language comprehension”, “attention/memory”, and “motor function”.

**Figure 1:**
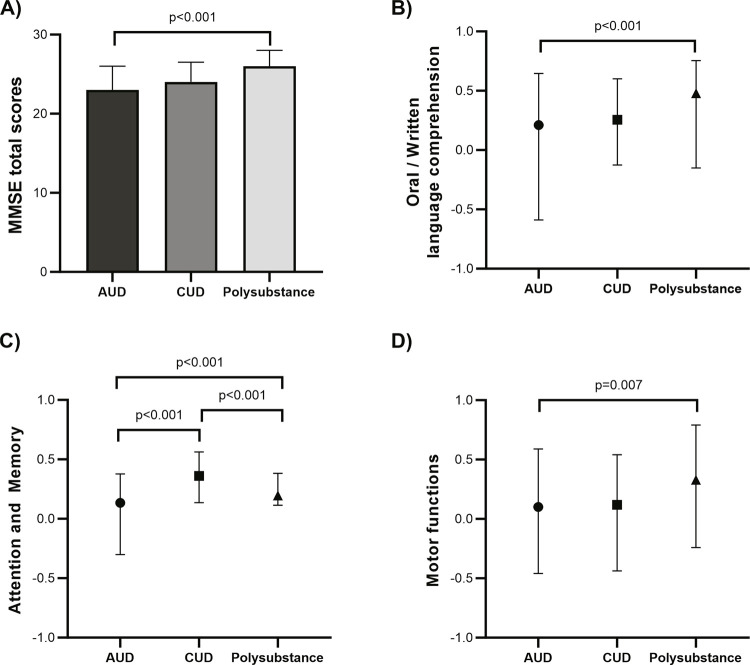
Mini Mental State Examination (MMSE) performance between substance use groups. (A) MMSE total score; (B) Oral and written language comprehension; (C) Attention and memory; (D) Motor functions. All p-values shown are adjusted for multiple comparisons (Dunn-Bonferroni post-hoc test). AUD = alcohol use disorder; CUD = cocaine/crack use disorder.

Total MMSE scores were significantly different between the three groups evaluated (χ^2^_[2]_ = 20.270, p < 0.001, with medians [IQR 25-75] of 23 [21-26] for AUD, 24 [22-27] for CUD, and 26 [23-28] for polysubstance use) ( Figure 1A ). Post-hoc comparisons indicate that individuals with AUD had worse performance than individuals with polysubstance use (p < 0.001). Individuals with CUD had similar MMSE scores to each of the other two groups (p > 0.05). Similar findings were observed when specific components related to MMSE performance were compared: individuals with AUD performed significantly worse than individuals with polysubstance use in oral/written language comprehension (χ^2^_[2]_ = 15.343, p < 0.001) ( Figure 1B ), and motor functions (χ^2^_[2]_ = 9.960, p = 0.007) ( Figure 1D ). Regarding attention/memory, all three groups were significantly different (χ^2^_[2]_ = 24.568, p < 0.001) ( Figure 1C ).

**Figure 2:**
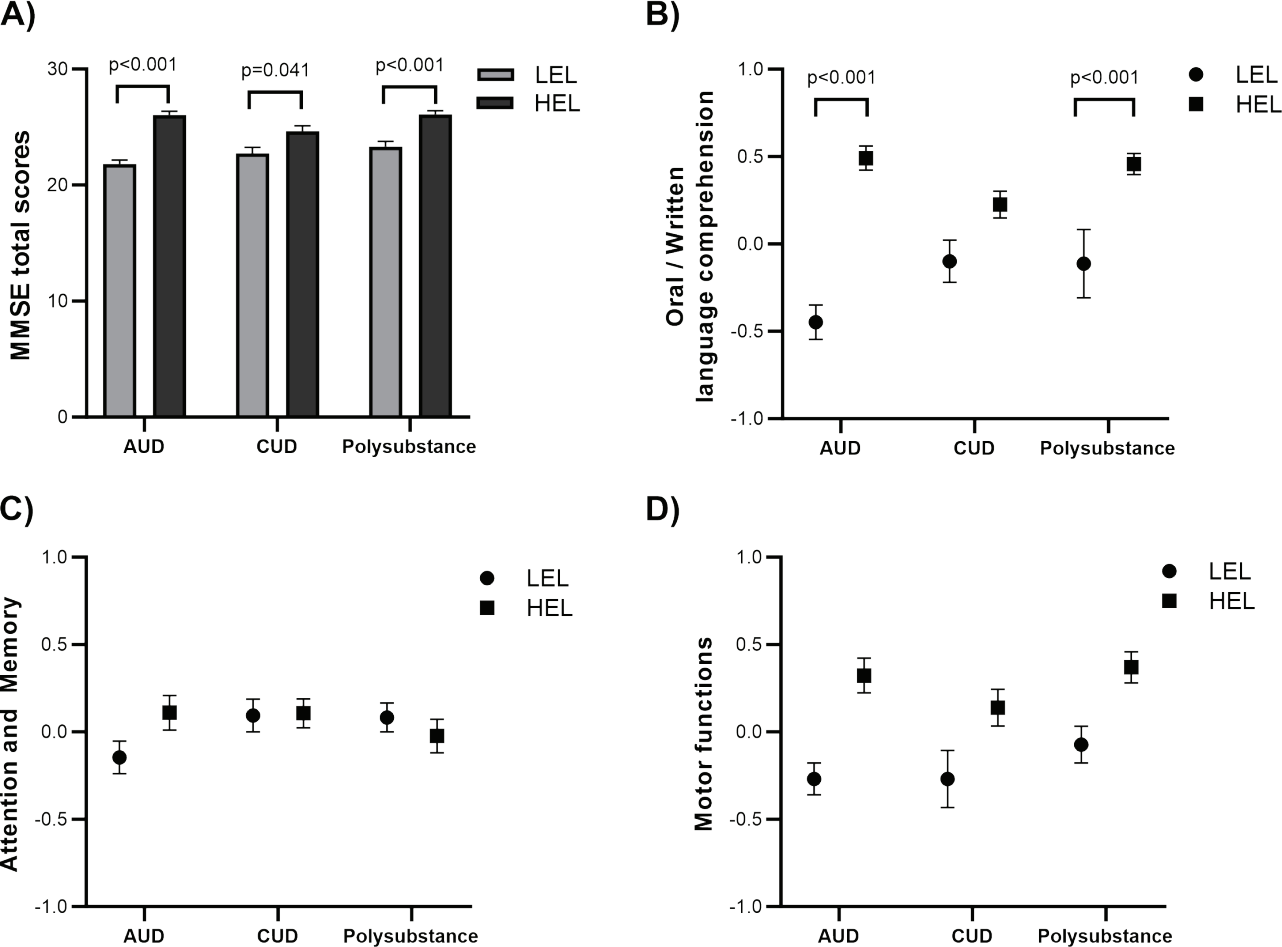
Moderating effect of educational level on Mini Mental State Examination (MMSE) performance according to substance use. (A) MMSE total score: pinteraction = 0.008. AUD + LEL differed from AUD + HEL p < 0.001, CUD + HEL p < 0.001, polysubstance + HEL p < 0.001, CUD + LEL differed from AUD + HEL p < 0.001, CUD + HEL p = 0.041, polysubstance + HEL p < 0.001, Polysubstance + LEL differed from AUD + HEL p < 0.001, polysubstance + HEL p < 0.001; (B) Oral and written language comprehension: pinteraction = 0.001, AUD + LEL differed from AUD + HEL p < 0.001, CUD + HEL p < 0.001, polysubstance + HEL p < 0.001, CUD + LEL differed from AUD + HEL p = 0.002, polysubstance + HEL p < 0.001, Polysubstance + LEL differed from AUD + HEL p < 0.001, polysubstance + HEL p < 0.001; (C) Attention and memory: pinteraction = 0.162; (D) Motor functions: pinteraction = 0.604. All p-values shown are adjusted for multiple comparisons (Bonferroni post-hoc test). AUD = alcohol use disorder; CUD = cocaine/crack use disorder; HEL = high educational level; LEL = low educational level.

Subsequent analysis, evaluating the possible moderating effect of educational level on MMSE performance, revealed a significant interaction between substance use and education status (Wald = 9.559, p_interaction_ = 0.008) ( Figure 2A ). In all groups evaluated, individuals with AUD and LEL performed significantly worse when compared to individuals with HEL, (p < 0.001). Similar findings were observed comparing individuals with CUD and LEL with all individuals with HEL (AUD p < 0.001, CUD p = 0.041, and polysubstance use p < 0.001). Moreover, polysubstance users with LEL had lower MMSE scores than individuals with HEL and alcohol use (p < 0.001) and polysubstance use (p < 0.001). Oral/written language comprehension composite scores showed similar findings (p_interaction_ = 0.001) ( Figure 2B ). Interaction effects were not significant for attention/memory or motor function scores (p_interaction_ = 0.162, and p_interaction_ = 0.604) ( Figure 2C and Figure 2D , respectively).

## Discussion

This study used a simple, user-friendly (easy training and rating), and widely validated test to investigate the effect of different substances (alcohol, cocaine and crack, and polysubstance use) – as well as the additional influence of educational level on cognitive performance. As far as we know, our study is the first to show a difference in MMSE scores detected among individuals who use distinct substances. Individuals with AUD presented worse cognitive performance than individuals with polysubstance use, especially in oral/written language comprehension and motor function. Attention and memory abilities differed between the three groups assessed, where the AUD group scored lowest, followed by polysubstance, and CUD groups. Further analyses revealed that low educational level was associated with lower MMSE scores, in all addiction groups, compared to a high educational level. This finding was more pronounced for oral/written language comprehension.

Overall, individuals with AUD had the most impaired performance, with lower scores for total MMSE and all of its subdomains that were identified in the PCA analysis. This finding partially supported our initial hypothesis, since individuals with polysubstance use did not present similar cognitive performance compared to individuals who used alcohol exclusively. However, we must point out that alcohol withdrawal may contribute to cognitive impairment during the first 2 weeks due to withdrawal symptoms and use of medication. A recent relevant editorial mentioned that it has become increasingly clear that biopsychosocial factors (e.g., sex, biological predisposition, sensation seeking, adverse childhood experiences, socioeconomic vulnerability, availability, etc.) are intrinsically associated not only with first use, but also with maintenance of polysubstance use and its consequences on cognitive dysfunction in the context of SUD. Therefore, the editorial suggested that SUD is part of a dynamic and complex system that the research field should embrace, precisely accounting for polysubstance use patterns that, in interaction with biopsychosocial factors, might lead to cognitive dysfunction.^31^

Few studies have assessed cognitive performance using the MMSE, but in all studies lower scores were reported in individuals with SUD – including alcohol, crack cocaine, opioids, and methamphetamine, compared to controls.^17 - 19^ Comparison between substances of preference has also been conducted, but similar MMSE performance was observed previously.^19 , 32^ It is important to emphasize that most previous studies had smaller samples than ours, which could explain the minor discrepancies between these specific findings. In addition, these studies differ from ours in relation to some sociodemographic and clinical characteristics – such as age, educational level, and abstinence period.

It is also relevant to mention that most studies do not differentiate between crack and cocaine, since few perform toxicological analyses to find other components besides the main psychoactive substance, which is cocaine itself. A systematic review has shown that 90% of the articles analyzed provided evidence of cognitive impairments involving inhibitory control in cocaine and/or crack users.^33^ They exhibited difficulties with cognitive processing, manifest in failures of emission, inhibition, and monitoring of responses during the execution of tasks designed to evaluate inhibition. Elevated levels of impulsiveness were also reported, but most studies did not contain clear information on the route of cocaine administration (whether inhaled, injected, or smoked as crack). They emphasize that although crack exhibits distinct patterns of consumption, route of administration, and potential for addiction when compared to cocaine, it can cause more severe cognitive and behavioral impairments than those exhibited by users of the inhaled form of this psychoactive substance. In this respect, this study corroborates the lack of studies investigating the specific clinical, cognitive, and behavioral characteristics of crack users.

Other evidence also showed that most individuals with AUD could have some cognitive or memory decline, even during long periods of abstinence.^7 , 34^ Moreover, a spectrum of neurocognitive disorders is frequently associated with alcohol addiction, from mild to major impairments,^28^ and includes alcohol-related dementia and Wernicke’s encephalopathy.^35^ Alcohol-related brain damage is linked to a series of pathophysiological processes, including thiamine deficiency, neuroinflammation, oxidative stress, alterations in liver enzymes and occurrence of liver diseases, and recurrent cycles of withdrawal and intoxication.^35^ Neuroimaging studies revealed that chronic and excessive use of alcohol are involved in structural alterations and functional damage, which might subsequently lead to cognitive impairments.^34 , 35^ Alterations in prefrontal and orbitofrontal cortex, nucleus accumbens, hippocampus, and cingulate cortex have been associated with alcohol abuse. These regions are also involved in the reward system, mechanisms of the dependence itself, executive functions, learning and memory.^34 - 36^ Altogether, this evidence corroborates the association of alcohol use with lower MMSE scores observed in our study.

The effect of educational level (measured by years of formal education) on MMSE scores was also detected in our analysis. However, this finding was equally observed across all substances, suggesting that this factor has a crucial and important effect on MMSE performance. Previous studies associated lower educational level with lower MMSE scores,^37^ and poor cognitive recovery over time in AUD users.^38^ In fact, the influence of educational level on cognitive performance is widely discussed in the literature, although most of the studies have been conducted in individuals with SUD in countries with high socioeconomic levels and HEL.^27^ Our study is one of the first to indicate the role of education on MMSE performance in SUD in a middle-income country with a relatively low educational level (8 years on average). On the other hand, the influence on MMSE scores of substance use characteristics, such as years of substance use and severity – or psychiatric comorbidities – was not detected. As our sample consists of patients undergoing detox with a severe clinical presentation, it is possible that the variation in these data does not have the potential to significantly affect the outcome, preventing us from detecting any association. Other studies also did not observe the influence of drug use profile and comorbidities on MMSE scores, corroborating our findings.^32^

It is important to consider some limitations of our study. First, the MMSE was the only tool administered and it does not evaluate executive functions,^35^ which is one of the most common cognitive impairments in addiction. However, this screening tool was able to detect differences between the groups of patients and the influence of educational level and also to infer some interesting findings from distinct MMSE domains. Also, we did not directly assess intellectual disabilities, which might influence the results observed. Second, our sample was assessed on the 2nd day after hospital admission and acute withdrawal symptoms could have affected some of the results, especially in alcoholics and polysubstance users. Third, it is relevant to mention the lack of a control group. Although it was not the goal of our study, this prevented us from analyzing and comparing the presence of more specific cognitive deficits in the sample. Fourth, the sample comprised men who sought treatment for addiction, and the results obtained may not represent the performance of other populations, such as women and users who do not seek treatment. Finally, although there is a significant difference in educational level between the three groups, the mean values are quite similar and may not result in a clinical discrepancy.

In summary, our study evaluated a large sample comprising three different groups of individuals with SUD (AUD, CUD, and polysubstance use). A slightly different pattern of cognitive impairments was observed according to the substance used. Our results also demonstrated the importance of schooling for cognitive performance. A routine screening instrument for cognitive function in individuals with SUD is vital and could be used to monitor these functions during treatment follow-up. The recovery of cognitive processes during abstinence and monitoring of lasting cognitive impairment may warrant other psychosocial strategies in addition to those routinely used during the long and hard process of addiction recovery.
